# Supplementary material for: Wire Electrodes Embedded in Artificial Conduit for Long-term Monitoring of the Peripheral Nerve Signal
Source: Micromachines (Basel). 2019 Mar 13;10(3):184. doi: 10.3390/mi10030184 (PMC6471311; doi:10.3390/mi10030184)
Supplement: Supplementary file 1 [file micromachines-10-00184-s001.zip › micromachines-458857- Supplymentary/Supplemantary Material- .docx]

**Supplementary Material: Wire Electrodes Embedded in Artificial Conduit for Long-term Monitoring of Peripheral Nerve Signal**

Woohyun Jung, Sunyoung Jung, Ockchul Kim, HyungDal Park, Wonsuk Choi, Donghee Son, Seok Chung and Jinseok Kim

**Figure S1.** (**A**) Pre-bent PI film for fixing the silicon tube onto the epineurium. (**B**) Fixed PI film onto the silicon tube using the gingival mask.

**Figure S2.** (**A**) Fixing the screws on the skull to increase the binding force. (**B**) Neck collar for preventing self-injury and headstage filled with gingival mask for preventing contamination.

**Figure S3.** Results of real-time neural signal measurement at an early stage.


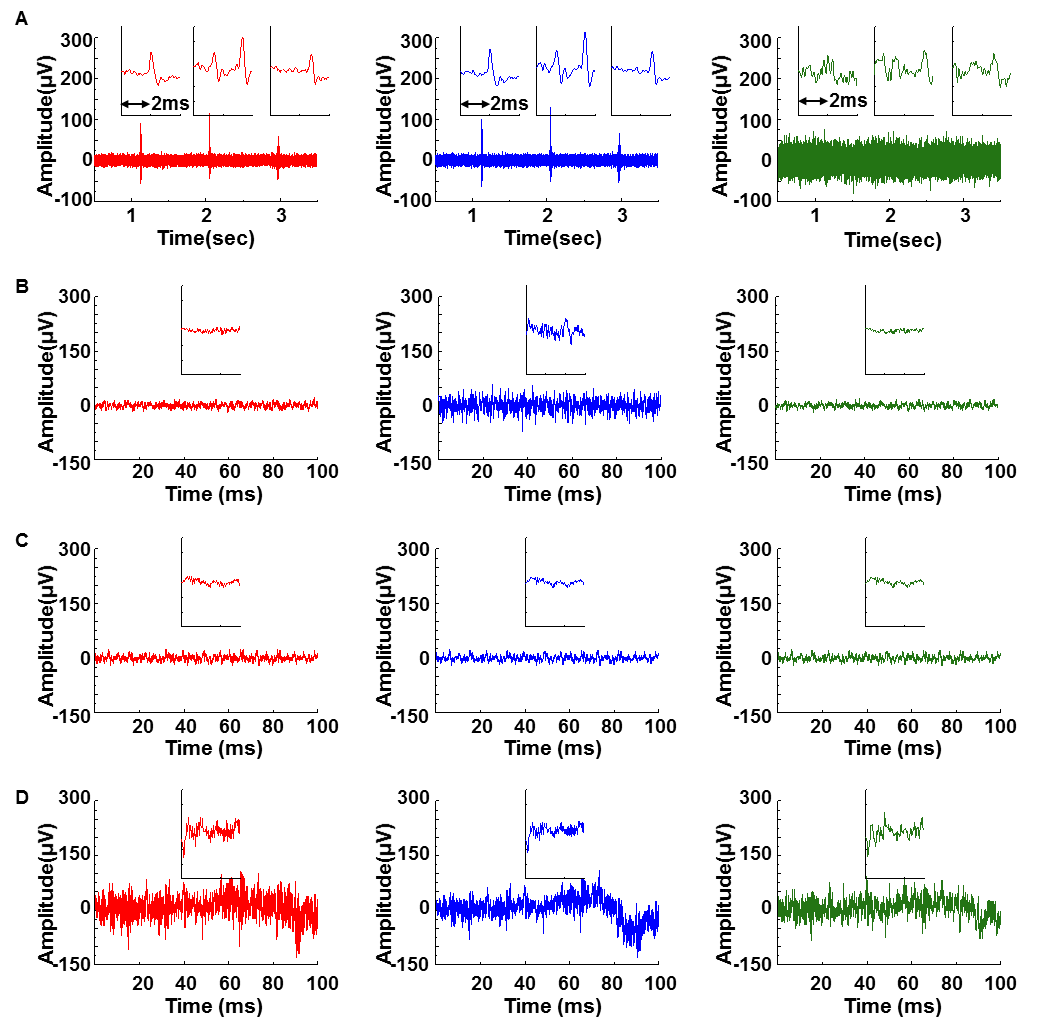


**Figure S4.** Long-term neural signal measurement in 4 rats after 19 weeks. (**A**) Neural signals obtained from two (left, red; center, blue) of three wires in the one among 4 rats. It was expected that the same nerve fibers would touch the two wire electrodes simultaneously as the nerve grows longitudinally and the electrodes were arrayed longitudinally. The noise level of the rest wire electrodes were higher than those of others due to its mechanical breakdown (right, green). (**B**–**D**) Each neural signal in other 3 rats was not observed. Such issues can be happened due to the above same reason. We also assumed that if the regenerated axons inside the artificial conduit cannot contact the wire electrodes, the neural signals cannot be measured due to the large gap between axons and electrodes.


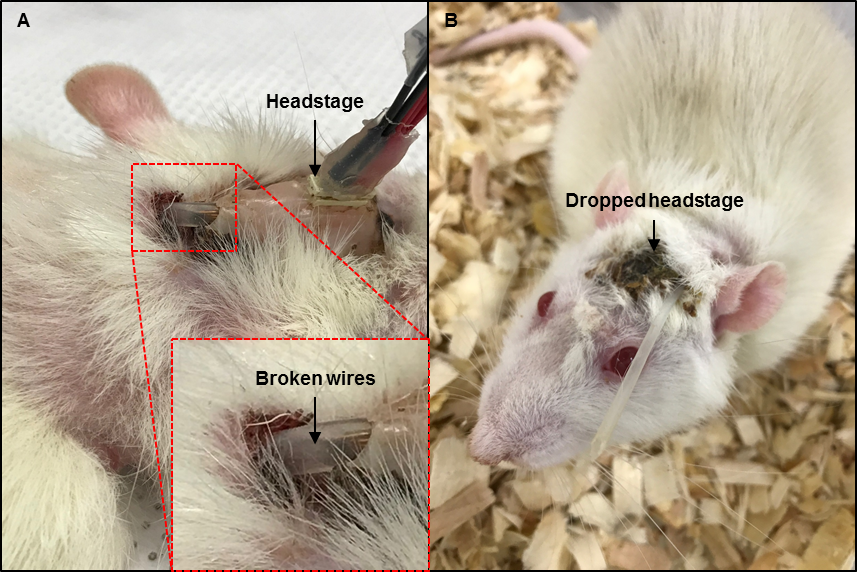


**Figure S5.** Mechanical breakdown issues in long-term implantation. (**A**) Breakdown of Cu interconnection cable inside the silicon tube (**B**) Delamination of the headstage mounted on rat’s head.
